# Endovascular strategies for post-dissection aortic aneurysm (PDAA)

**DOI:** 10.1186/s13019-020-01331-8

**Published:** 2020-10-01

**Authors:** Zhaoxiang Zeng, Yuxi Zhao, Mingwei Wu, Xianhao Bao, Tao Li, Jiaxuan Feng, Rui Feng, Zaiping Jing

**Affiliations:** Department of Vascular Surgery, Changhai Hospital, Navy Medical University, 168 Changhai Road, Shanghai, 200433 People’s Republic of China

**Keywords:** TEVAR, Post-dissection aortic aneurysm, False lumen, Endovascular repair, Candy-plug

## Abstract

Residual patent false lumen (FL) after type B aortic dissection (TBAD) repair is independently associated with poor long-term survival. Open surgery and endovascular repair result in good clinical outcomes in patients with AD. However, both treatments focus on proximal dissection but not distal dissection. About 13.4–62.5% of these patients present with different degrees of distal aneurysmal dilatation after primary repair. Although open surgery is the first-choice treatment for post-dissection aortic aneurysm (PDAA), there is a need for high technical demand since open surgery is associated with high mortality and morbidity. As a treatment strategy with minimal invasion, endovascular repair shows early benefits and low morbidity. For PDAA, the narrow true lumen (TL), rigid initial flap and branch arteries originating from FL have increased difficulties in operation. The aim of endovascular treatment is to promote FL thrombosis and aortic remodeling. Endovascular repair includes intervention from FL and TL sides. TL intervention techniques (parallel stent-graft, branched and fenestrated stent-graft among others) have been proven to be safe and effective in PDAA. Other FL intervention techniques that have been used in selected patients include FL embolization and candy-plug techniques. This article introduces available endovascular techniques and their outcomes for the treatment of PDAA.

## Introduction

Thoracic endovascular aortic repair (TEVAR) is widely used for type B aortic dissection (TBAD) due to the availability of advanced endovascular techniques and acceptable outcomes. Currently, the treatment strategy of TEVAR is to enhance aortic remodeling as it excludes proximal tear, reduces blood pressure and induces thrombogenesis in the false lumen (FL) [1]. However, backflow from distal entry tears maintains perfusion to FL, inducing aneurysm formation. About 13.4–62.5% of patients show different degree of distal aneurysmal dilatation after TEVAR [2, 3]. The presence of distal residual tears affects the long-term survival of patients [4]. Although open surgery is still the first-choice treatment for post-dissection aortic aneurysm (PDAA), it still requires high technical demands and is also associated with increased mortality and morbidity [5]. Endovascular strategies regarded as less invasive treatment with good early outcomes are suitable for patients unable to tolerate open surgery. The indications for endovascular treatment are similar to those for open surgery, such as aneurysms > 5.5 cm in diameter or those with rapidly expand > 1 cm/year and aneurysm-related symptoms (refractory pain, malperfusion and compression complications, et al.)*.* Nowadays, available endovascular methods are increasingly used in the treatment of PDAA in high-volume centers. Several electronic databases (including EMBASE and MEDLINE) were searched to identify potential studies. And searching strategies included “TEVAR”,“post-dissection aortic aneurysm”, “chronic aortic dissection”, “endovascular repair” and “stent-graft”. According to the relevant results, we can divide the techniques into interventions from FL side and from True lumen (TL) side. This article aims to discuss different endovascular strategies and outcomes for PDAA.

## Trans-TL repair

### TEVAR

The concept of TEVAR is excluding proximal entry tears to induce positive aortic remodeling. As to PDAA, TEVAR extends the tubular stent-grafts above the level of celiac axis. This can exclude tears above the celiac trunk and reduce flow and pressure of the FL. However, distal residual tears may affect long-term survival. Studies have demonstrated that aortic remodeling is mainly concentrated in the covered thoracic aortic segment after TEVAR [6]. A report by Mani K et al. indicated that complete FL thrombosis after TEVAR for chronic AD occurred in one-third of patients. But for most cases, positive aortic remodeling and complete thrombosis were frequently limited to the thoracic aortic segment [7]. The latest meta-analysis from Boufi M showed that when chronic AD is treated by TEVAR, the 1-year and 3-year follow-up survival rates were similar, which was 91%, and aortic-related mortality was 3.2%. The reintervention rate was 20.2%, and the risk of aortic rupture was 3% [8]. In cases of PDAA extent to visceral segment or abdominal aorta, this method is confined to exclude all tears. A survey from our center reported that the average number of residual tears was 6.10 ± 3.16. Among them, the numbers in thoracic descending aorta, visceral segment, infra-renal abdominal aorta and iliac artery were 1.07 ± 1.59, 1.53 ± 1.27, 2.30 ± 1.41 and 1.20 ± 0.80, respectively [2]. The most drawback of traditional TEAVR is that it is limited to exclude the entry tears in thoracic descending aorta.

**Fig. 1:**
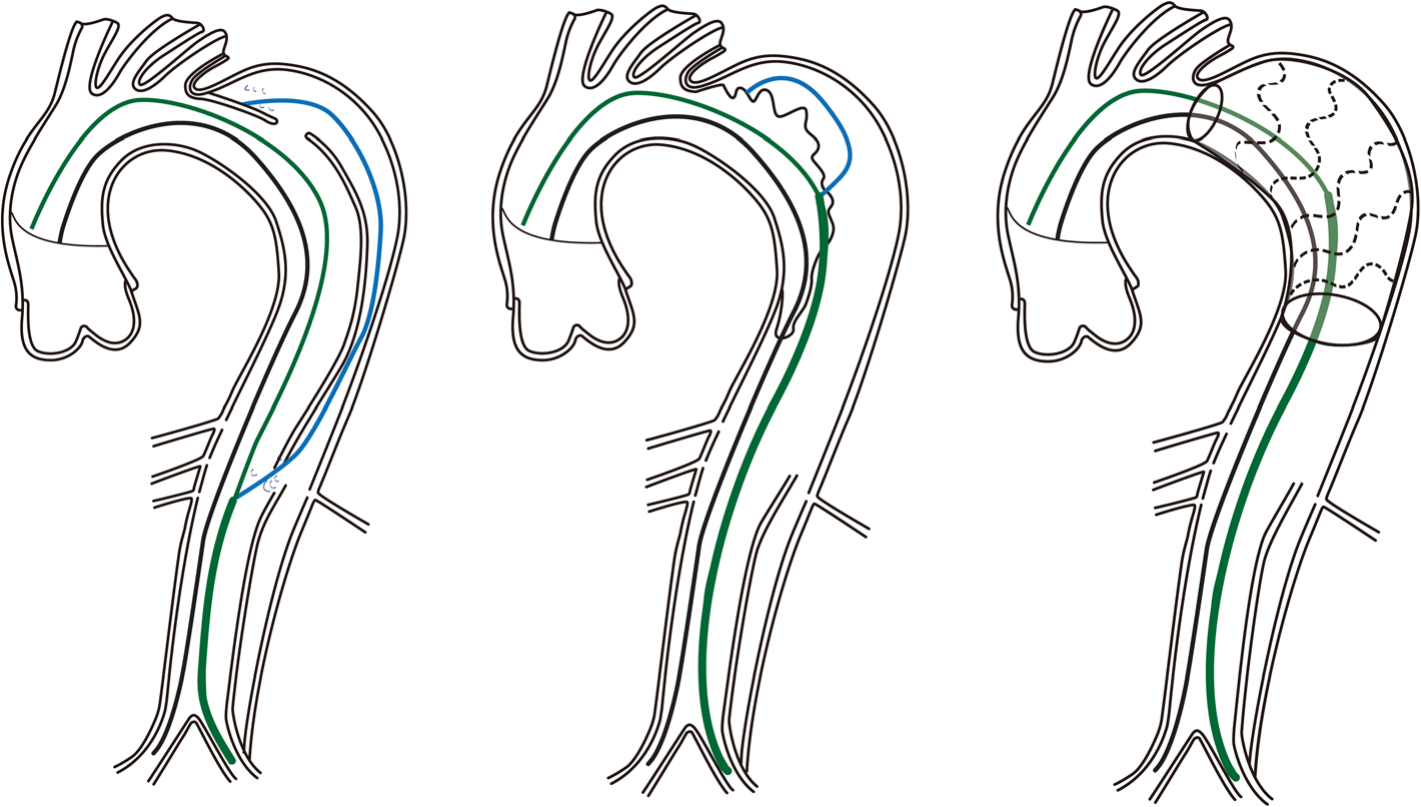
Endovascular scissors technique

The importance of the distal landing zone for TEVAR in the treatment of aortic dissection has been highlighted in previous studies. The “cheese wire” technique describes that using a hydrophyllic guidewire to unzip initial flap creates a landing zone for endovascular treatment. The method was first used by Watkinson in a patient with severe iliac artery occlusion [9]. Later on, Iwakoshi S et al used this method to treat three patients with chronic AD, and there was one case for type III endoleak and another for renal artery occlusion which required re-intervention [10]. However, significant risks with this technique persisted, for example, intimal flap occlusion of visceral arteries. Some scholars have also explored the use of endovascular scissors in AD [11, 12]. Endovascular scissors have been developed by Alomran F for complicated acute TBAD with severe malperfusion (Fig. 1). The technique was performed in three patients and no complications occurred at 2-year follow-up [13]. Although these techniques can create a complete distal landing zone, available information is case series. Besides, the safety and generality of these techniques remain a problem to explored.

### The evolution of PETTICOAT (provisional extension to induce complete attachment) technique

**Fig. 2:**
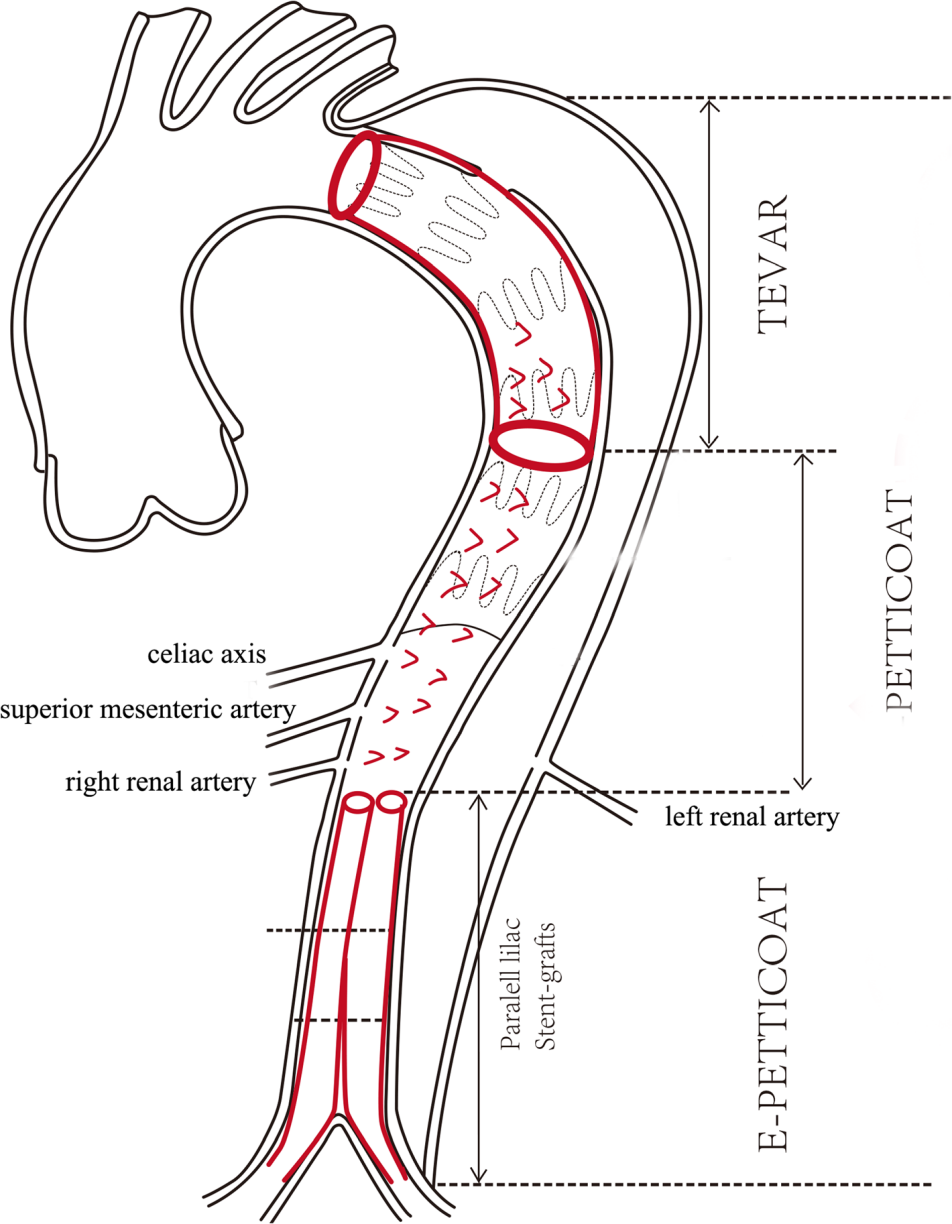
PETTICOAT technique

The PETTICOAT concept involved a standard TEVAR to exclude proximal tears and distal uncovered stent-graft to expand distal TL associated with stabilization of dissecting initial flap. Therefore, this method was more effective in non-chronic dissection, because initial flap can be expanded. It evolved from the earliest prototype of a simple bare stent-graft to a composite device design. The prototype of PETTICOAT technique dates back to 2003 (Fig. 2). The first report on the use of self-expanding bare stents for the treatment visceral arteries malperfusion was by Ito N et al [14]. In 2006, this technique was officially named by Nienaber CA [15]. Twelve patients demonstrated distal TL collapse and a perfused FL after sealing of the proximal tears were treated. A stent-graft was placed for distal extension for the stent-graft previously implanted to obliterate sustained abdominal FL flow and pressurization. After one-year follow-up, 11 patients had complete thoracic thrombosis with benign aortic remodeling. One patient without FL thrombosis died at 11 months due to aortic rupture. Later, Lars Kock introduced DEEB PETTICOAT (Distal Extended Branched PETTICOAT) technique which worked in combination with FL arteries construction to treat 26 patients with complicated TBAD (unpublished data, presented at VEITH Symposium 2017). There was no aortic related deaths or branched arteries occlusion within 28.7 months after surgery. Besides, Kazimierczak A tried the extended petticoat strategy in TBAD [16]. This strategy provided diffuse mechanical aorto-iliac support and led to complications necessitating re-intervention when treating extensive TBAD. Based on the concept of PETTICOAT, Lombardi JV et al. proposed a composite device design to treat complicated type B aortic dissection [17]. The device comprised a pathology-specific device comprising a proximal stent graft with barbs and a distal bare stainless steel stent. The STABLE I trial recruited 86 patients (55 in acute phase and 31 in nonacute phase). The 30-day mortality rate was 5.5% (3 of 55) for acute and 3.2% (1 of 31) for nonacute. There was an overall increase in true lumen diameter, and false lumen shrinkage was observed both in acute and nonacute phase at the level of thoracoabdominal aortas. At 5 years, freedom from intervention was 65.5% ± 7.5% for acute and 71.2% ± 9.0% for nonacute patients. However, some scholars were worried about the risks associated with aneurysm formation since the technique expanded the TL leaving distal tears untreated [18, 19]. More robust evidence was needed to demonstrate the safety and effectiveness of the technique.

### STABILISE (stent-assisted balloon-induced intimal disruption and relamination in aortic dissection repair) and Knickbocker technique

**Fig. 3:**
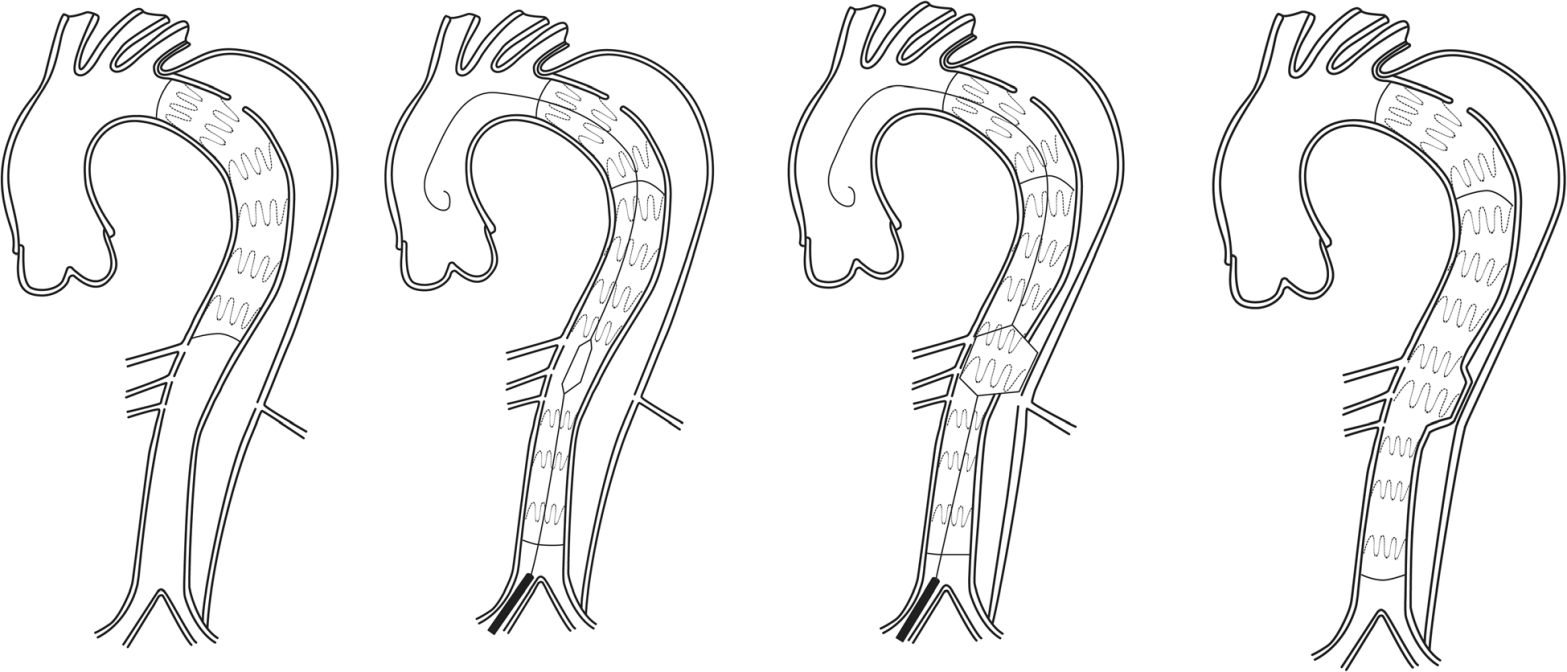
Knickbocker technique

Different from PETTICOATS technique, STABILISE and Knickbocker technique worked by deliberately disrupting initial flap to create a single lumen. After operation, the distal backflow was blocked. In 2014, Hofferberth SC et al. proposed the STABILISE technique derived from PETTICOAT [20]. Eleven patients underwent proximal descending aortic endograft combined with stent-assisted balloon of the thoracoabdominal aorta to treat complicated AD. There were no intraprocedural complications recorded. However, a patient died within 1 month, although complete FL obliteration was achieved in nine patients. The group of Faure EM has also reported experience in treating TBAD whereby in a series of 41 patients, no intra-procedural complications were recorded. Exactly 15 branch arteries originating from FL were constructed. The 30-day death, stroke and paralysis/visceral ischemia rates were 2, 0, 5, and 2% respectively. The patency of visceral branched arteries was 93%. Eight patients required secondary intervention. No aortic related deaths occurred. Complete FL obliteration and aortic remodeling was realized in all patients at the thoracoabdominal level [21]. In addition, the technique was successfully applied in patients with Marfan syndrome [22]. All patients had complete aortic remodeling of the treated aortic segment. Knickbocker, another technique for intimal flap disruption, was described by Kölbel T in 2014 [23]. With this technique, an oversized thoracic tubular endograft was placed in the narrow TL to induce intimal flap disruption, which occluded distal backflow into FL. It has been successfully used in three patients with PDAA, and the FL aneurysm remained thrombosed with no mortality recorded during follow up (Fig. 3). Both STABILISE and Knickbocker technique were not widely accepted due to the potential risk of aortic rupture.

### Parallel stent-graft technique (PST, Fig. 4)

PST is widely used to treat complex aortic lesions [24, 25]. Because of the low requirement for stent-grafts, the technique owes unique advantages in dealing with emergent cases. The technique was applied by Liu J et al. to treat 21 post-dissection thoracoabdominal aneurysms [26]. Four cases of type I endoleaks occurred after repair, which disappeared spontaneously within 1 month. Neither spinal cord ischemia nor organ malperfusion were observed. During an average follow-up of 16 months, two cases of type II endoleaks occurred. Nineteen FL achieved complete thrombosis, and the total aortic diameter decreased from 57.3 ± 8.4 to 55.3 ± 7.4 mm. Three (4.3%, 3/70) target branch arteries occluded. To avoid the risk of endoleak, sufficient overlap between stents was recommended. How to “maintain the balance between sufficient overlap and cost” remained to be explained. Furthermore, although the results of the study were encouraging, the long-term patency of visceral branches remained a concern.

**Fig. 4:**
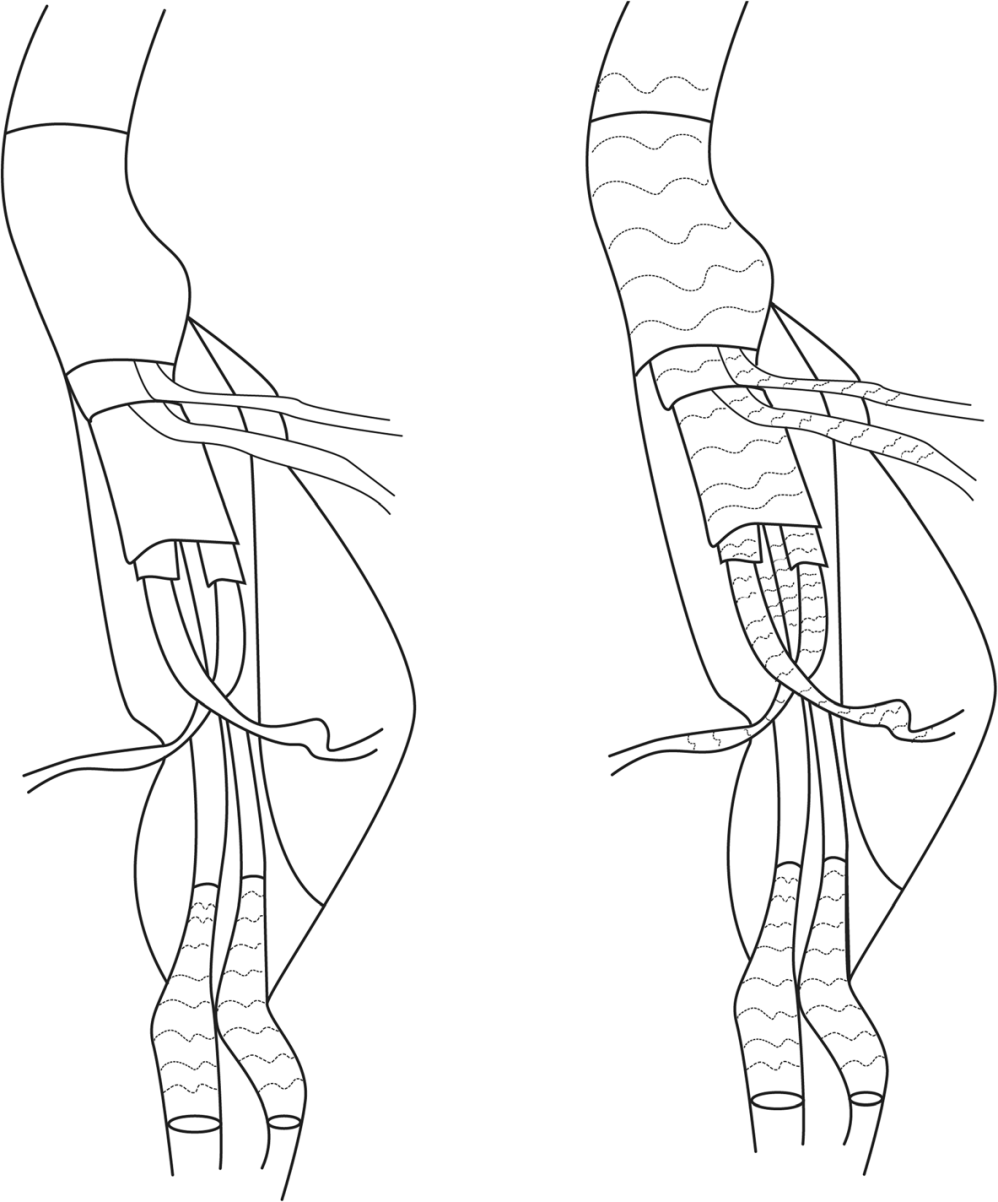
Parallel stent-graft technique

### Branched and fenestrated stent-graft (Fig. 5)

Branched and fenestrated stent-graft is considered a safe and effective way of treating thoracoabdominal aortic aneurysms (TAAA) [27, 28]. In cases where TAAA has appropriate anatomical conditions, branched and fenestrated stent-graft is taken as the first-line treatment option [29]. Unlike degenerative TAAA, the anatomies of PDAA have some drawbacks. The narrow TL, branch arteries that arise from FL and stiff initial flap limit the effect of endovascular treatment. Upon the expansion of narrow TL, the anatomical characteristics of branch arteries change accordingly. The fenestrated stent-graft can adapt to the relatively narrow TL, but super-selection of branch arteries places great technical difficulties on the operators. The existence of small cuff to branched stent-graft is important in reconstructing visceral branched arteries but requires a spacious room. Mayo Clinic applied auxiliary preloaded guidewires to treat TAAA on 83 patients with TAAA or pararenal aortic aneurysm. The technical success rate was 99.7% on average operation time of 160 min, 30-day mortality was recorded as 0% [30]. The result revealed that this technique could facilitate visceral branched arterial reconstruction and reduce the difficulty of operation. The concept of inner branched stent-graft provides an option for visceral arteries unsuitable for branched and fenestrated stent-graft. Findings from Katsargyris A et al. reported their early experience in treating complex aortic lesions with inner branched stent-graft. The 30-motality rate was 3.1% (1/32), and 1-year overall survival rate was 80%. During the follow-up period, four renal inner branches occluded in three patients. The patency of inner branched stent-graft at 1 year was 91.9 ± 4.5%, and re-intervention at 1-year rate was 11.6% ± 8.9% [31]. Another vexing problem was reconstruction of visceral artery, especially those totally originated from FL. Catheterization usually was placed from the TL, through the tear in the visceral segment and FL, into the target artery. Some scholars performed an in-situ fenestration to create a new approach to target vessels if there was no re-entry tear at the level of target vessels.

**Fig. 5:**
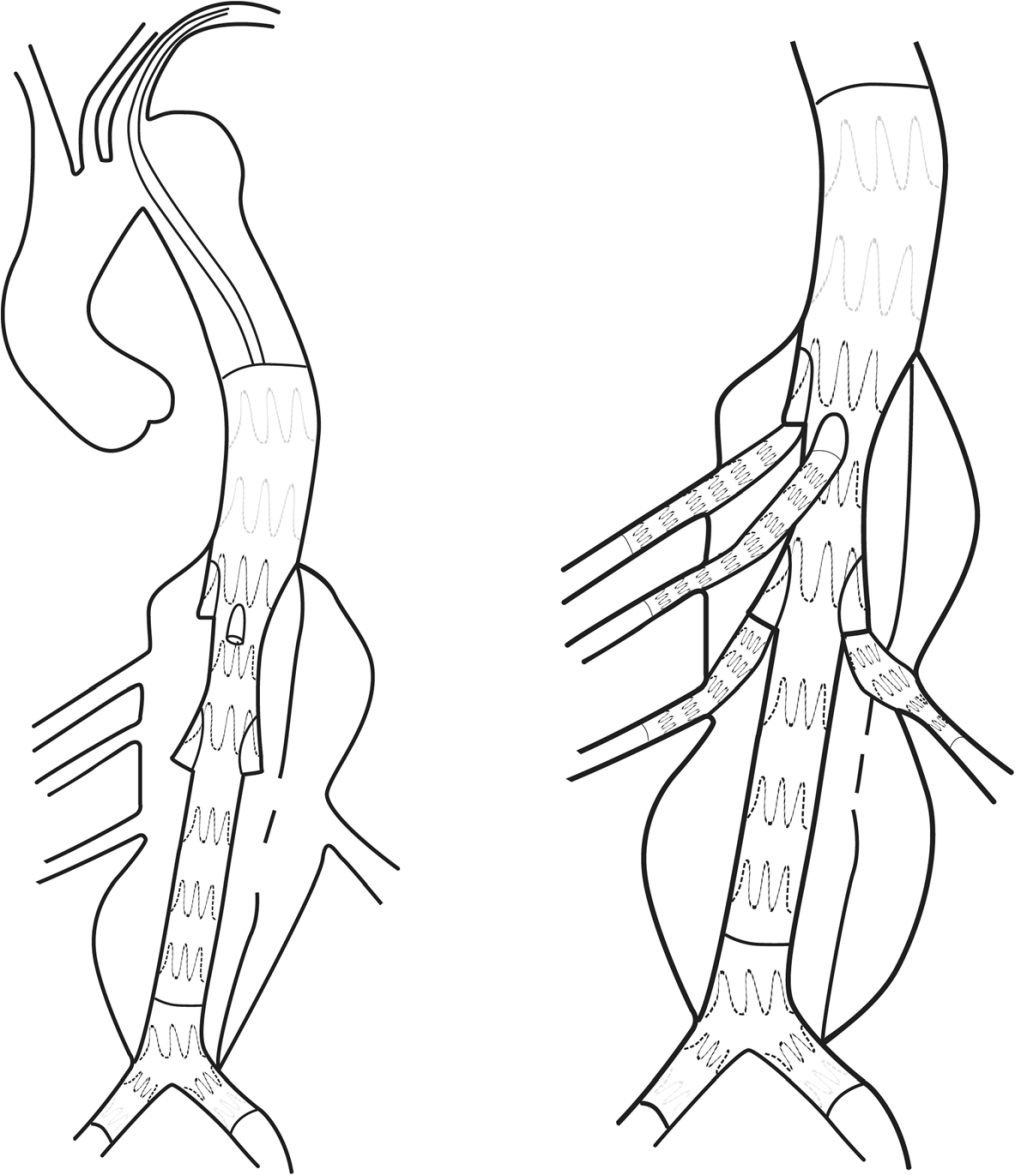
Branched and fenestrated stent-graft technique

### Multilayer bare stents/multilayer flow modulator (MBS/MFM)

A lot of controversies exist with the treatment of aortic lesions using MBS/MFM. MBS/MFM device induces thrombus formation as it changes the flow status in the FL. It sufficiently restores flow perfusion, decompress the FL, eliminate local flow recirculation, and reduces wall shear stress distribution. Moreover, the device restores flow to organ perfusion despite its effect on TL patency [32]. In a study, 38 patients having chronic AD were treated using the device. All-causes survival was 85.3% within 1-year follow up. No aortic ruptures or aortic-related deaths were observed. Aortic morphologic remodeling is reflected by a reduction in longitudinal length of the dissected aorta and FL volume [33]. In our experience, the technique was addressed to six cases with degenerative thoracoabdominal aortic aneurysms [34]. During a mean follow-up of 14 months, four patients realized aneurysm shrinkage, and two aneurysms maintained stabilization. Notably, the presence of MBS/MFM increased the operation difficulty if patients need re-intervention. The coil had to get through stent shunts to visceral artery, and the manoeuvrability of the stent was greatly reduced.

## Trans-FL repair

### FL embolization

FL embolization prevents backflow at the level of the distal descending aorta. Intervention from the FL side prevents excessive coverage of the aorta and reduces the risk of spinal cord ischemia. Currently, there are no devices designed for FL embolism. Coils, onyx glue and vascular plug are commonly used. In 2003, Loubert MC named the technique “the cork in the bottleneck” and successfully rescued two patients with PDAA [35]. Hofferberth introduced the concept of aortic FL thrombosis induction through embolotherapy (AFTER). Ten patients (four acute type A AD, three acute TBAD and three chronic TBAD) received staged aortic repair. Averagely within 7 months after initial repair, directed embolization was performed. Nine out of ten patients realized aneurysm shrinkage or stability. One patient with chronic AD died 4 months after post-embolization [36]. Another embolization device, iliac occluders, has also been reported [37, 38]. Kim TH compared the outcomes of patients who underwent TEVAR with/without FL procedure. No statistically significant difference in the extent of aortic remodeling (TL expansion and FL regression) was observed between two groups. The cumulative proportion of thoracic FL thrombosis was high in the group of TEVAR with FL procedure [39]. In our opinion, FL embolization was considered to be a complementary and auxiliary therapeutic measure, especially in the cases with great FL in the distal aorta.

### Candy plug (Fig. 6a)

**Fig. 6:**
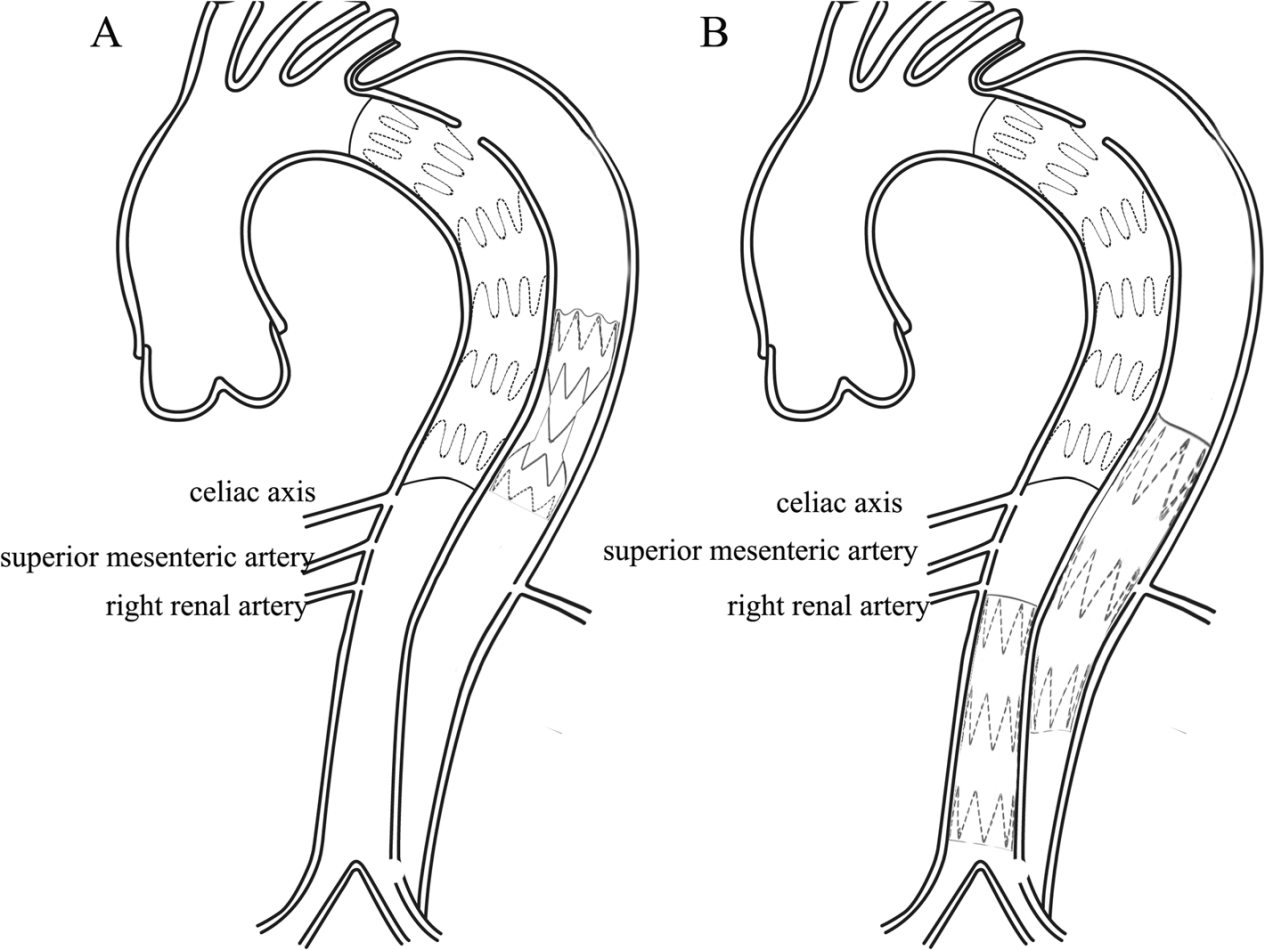
**a** Candy-plug technique; **b** “Double splint” technique

In addressing the problem with large false lumen diameters, Kölbel T created an extra-large vascular plug to occlude FL in chronic AD. A 42 mm diameter Zenith thoracic stent-graft was modified into a “wrapped candy” and deployed in the FL above the celiac axis level. The first-generation was sutured in the middle to create a central opening for locally available vascular plug devices [40]. In 2017, Rohlffs F reported the early outcomes of this technique in 18 patients. The clinical success rate was 94%. There were no intraprocedural complications. Complete distal FL occlusion was present in 15 patients [41]. The use of new generation Candy-Plug II in the treatment of 14 chronic dissections had been presented by Eleshra A. The new generation Candy-Plug II with a self-closing channel construction obviates additional placement of a plug. Its technical success was 100% with immediate FL flow completely blocked in 12 patients. Two patients received re-intervention. Eight of the nine patients presented aortic remodeling, while one aneurysm sac was stable [42]. Relevant anatomy for the use of the Candy-Plug Technique includes access to FL at distal aorta and sufficient extension of FL above the celiac axis for device deployment. By contrast, we deployed stent-grafts in FL to exclude tears from the side of FL in visceral segment and used TL stent-graft to exclude the tears and expand TL in infra-renal segment. The distal end of FL stent-graft was extended to abdominal segment (Fig. 6b). Eleven patients with PDAA were successfully treated from FL side. All patients had a stable or shrinking trans-aortic diameter in the abdominal aorta. There was no aortic related death during the follow-up. Wu IH et al used the iliac limb instead of suture to restrict the diameter in the middle section of stent-graft [43]. Another improvement of this technique had also been reported [44].

### Other intervention methods from FL

This procedure is only limited to some cases with severely narrow TL. In 2016, Bertoglio deployed branched stent-grafts in the TL and FL to exclude a PDAA. The right renal artery was reconstructed through the proximal TL. The branched stent-graft in the FL restored the other visceral arteries. Follow-up results after 6 months from operation showed that all visceral arteries were patent [45]. A report presented by Guo W et al. indicated successful total EVAR via retrograde reconstruction of the left renal artery from the false lumen [46]. On the one hand, these reports offered a new treatment idea: reconstruction of visceral arteries from FL could be applied in selected cases. On the other hand, these could not be considered as a standard of case.

## Conclusion

Stent-graft treatment in chronic type B dissection differs from acute type B pathology based on increased stiffness of the dissecting lamella and a continued FL expansion. Stent-graft deployment in chronic dissection does not necessarily focus on the expansion of TL, but aims to depressurize the FL by promoting progressive thrombosis. Although there are a series of methods to treat patients with PDAA, available clinic reports are limited to a few high-volume centers. Considering the advantages and disadvantages of each technology (Table 1),treatment recommendations are not only based on levels of evidence but the cost-effect analyses. Additionally, the complex anatomy of PDAA indicates that all endovascular strategies should be used with caution and strict indication. Personally, for those with TL highly stenosis, the future direction may reconstruct the visceral arteries in combination with the TL and the FL, so as to obtain the best hemodynamics and long-term prognosis. More prospective and randomized clinical trials are warranted to provide solid evidence on this topic.

**Table 1:** Summary of treatment strategies of PDAA

	Advantages	Disadvantages
Trans-TL Repair		
TEVAR	•Exclude tears above the celiac trunk •Reduce flow and pressure of the FL	•unable to exclude the entry tears in visceral artery segment of abdominal aorta •Distal residual tears may result in negative remodeling
PETTICOAT	•Expand distal TL •Stabilization of dissecting initial flap •Effective in non-chronic AD	•The risk aneurysm formation •Distal residual tears untreated
STABILISE and Knickbocker	•Create a single lumen to block distal backflow	•The risk of aortic rupture
Parallel stent-graft technique	•Flexible combination •Suitable for a variety of anatomy •Suitable for emergency and selective operation	•Endoleak •Recurrent aortic dissection •Chimney stent occlusion •Cost expensive
Branched and fenestrated stent-graft	•Suitable for uncomplicated anatomical conditions •Widely used in PDAA	•Difficult in TL stenosis cases •Difficult in reconstruction of visceral artery totally originated from FL.
MBS/MFM	•Restores flow perfusion •Decompress the FL	•Visceral artery ischemia •Difficulty in re-intervention
Trans-FL Repair		
FL embolization	•Avoid excessive coverage of the TL of aorta •Reduces the risk of spinal cord ischemia •A complementary and auxiliary therapeutic measure	•No special devices for FL embolism
Candy plug	•Promote the thoracic aorta segment aortic remodeling	•No effect for abdominal FL •The potential risk of aortic rupture

*PDAA* Post-dissection aortic aneurysm, *TL* True lumen, *TEVAR* Thoracic endovascular aortic repair, *FL* False lumen, *PETTICOAT* Provisional extension to induce complete attachment, *AD* Aortic dissection, *STABILISE* Stent-assisted balloon-induced intimal disruption and relamination in aortic dissection repair, *MBS/MFM* Multilayer bare stents/multilayer flow modulator

## Data Availability

Not applicable.

## Abbreviations

PDAA: Post-dissection aortic aneurysm
AD: Aortic dissection
TBAD: Type B aortic dissection
TEVAR: Thoracic endovascular aortic repair
TL: True lumen
FL: False lumen
